# Neuromyelitis Optica Spectrum Disorder Presenting With Longitudinally Extensive Transverse Myelitis and Pontine Involvement in a Patient With Systemic Lupus Erythematosus

**DOI:** 10.7759/cureus.97392

**Published:** 2025-11-20

**Authors:** Sally Hamad, Gagan Suresha, AbdulRahman Al-Mohammed

**Affiliations:** 1 General Medicine, Peterborough City Hospital, Peterborough, GBR; 2 General Medicine, Novosibirsk State University, Novosibirsk, RUS

**Keywords:** eight and a half syndrome, in-vitro fertilization (ivf), longitudinally extensive transverse myelitis (letm), neuromyelitis optica spectrum disorder (nmosd), systemic lupus erythematosus

## Abstract

Neuromyelitis optica spectrum disorder (NMOSD) is an autoimmune demyelinating condition of the central nervous system that may coexist with systemic lupus erythematosus (SLE), complicating diagnosis and management. We describe a woman in her 40s with long-standing SLE who presented with acute urinary retention, bilateral lower limb weakness, and diplopia following a febrile illness after unsuccessful in-vitro fertilization (IVF). Initial assessment suggested urinary tract infection, but rapid neurological deterioration prompted MRI, which revealed longitudinally extensive transverse myelitis (LETM) extending from T9 to the conus medullaris and pontine lesions with contrast enhancement. Cerebrospinal fluid analysis showed marked lymphocytic pleocytosis, and the diagnosis of NMOSD was favored. The patient received high-dose intravenous methylprednisolone followed by transfer for plasma exchange (PLEX). This case highlights the diagnostic overlap between SLE and NMOSD and emphasizes the importance of early recognition, imaging, and immunosuppressive therapy to prevent irreversible neurological sequelae.

## Introduction

Systemic lupus erythematosus (SLE) is a chronic multisystem autoimmune disease characterized by widespread immune complex deposition and inflammation affecting multiple organs. Neurological involvement, termed neuropsychiatric SLE, occurs in up to 40% of patients and encompasses a broad range of central and peripheral nervous system manifestations, including seizures, psychosis, and transverse myelitis [1]. Transverse myelitis is one of the most severe and potentially disabling neurological complications of SLE, often resulting in significant morbidity if not recognized and treated promptly [2].

Longitudinally extensive transverse myelitis (LETM), defined as an inflammation of the spinal cord extending across three or more vertebral segments on MRI, often resulting in severe motor and sensory deficits, has been increasingly associated with neuromyelitis optica spectrum disorder (NMOSD) [3]. NMOSD is an autoimmune disease that primarily targets the optic nerves and spinal cord, causing episodes of vision loss and paralysis. It is driven by antibodies that attack astrocytes, the supportive cells of the central nervous system. While NMOSD may occur independently, its coexistence with systemic autoimmune diseases such as SLE has been reported, suggesting overlapping immunopathogenic mechanisms [4]. The presence of LETM in a patient with established SLE warrants evaluation for NMOSD, as both the treatment approach and long-term prognosis differ from that of lupus myelitis [5].

Hormonal and immunological factors may influence disease activity in both SLE and NMOSD. Assisted reproductive treatments, such as in-vitro fertilization (IVF), and recent pregnancy events have been recognized as potential triggers for autoimmune flares through hormonal modulation and immune activation [6]. Early differentiation between lupus myelitis and NMOSD is therefore critical, as delayed recognition may lead to irreversible neurological deficits. Here, we report a rare case of SLE complicated by LETM and pontine involvement following IVF and medical pregnancy termination, representing an overlap presentation of NMOSD in a patient with lupus.

## Case presentation

A woman in her 40s with known SLE (involving joints, kidneys, and eyes), hypertension, and uterine fibroids presented to the emergency department with acute urinary retention and bilateral leg weakness. She had undergone medical termination of pregnancy following a failed IVF attempt approximately 10 days earlier and reported two weeks of low-grade fever and dry cough. Initially, she was diagnosed with a urinary tract infection and discharged with antibiotics; however, she re-presented with rapidly progressive weakness, paresthesia, and diplopia.

On examination, she was afebrile and alert. Neurological assessment revealed flaccid paraparesis (power 0/5 at hips, 1-2/5 at knees, and 0/5 at ankles), absent lower limb reflexes, and preserved upper limb strength. Sensation was intact. Cranial nerve examination showed ophthalmoplegia with impaired abduction and adduction of both eyes, right facial weakness, horizontal and vertical nystagmus, and diplopia consistent with 8½ syndrome. There was no neck stiffness or altered mental status.

Laboratory investigations (Table 1) demonstrated lymphopenia (0.4×10^9^/L), hyponatremia (131 mmol/L), elevated erythrocyte sedimentation rate (ESR) (130 mm/h), normal CRP (6 mg/L), and stable renal function (creatinine 117 µmol/L).

**Table 1:** Initial laboratory investigations This table summarizes the patient’s baseline laboratory results at presentation, including hematological, biochemical, and inflammatory markers. It highlights significant abnormalities such as lymphopenia, hyponatremia, and markedly elevated ESR, while CRP remained within normal limits. These findings provide supportive evidence of systemic inflammation and immune dysregulation in the context of SLE and suspected NMOSD. ESR: Erythrocyte Sedimentation Rate; CRP: C-reactive protein

Parameter	Result	Units	Reference Range
Lymphocytes	0.4	×10⁹/L	1.4 – 4.8
Sodium	131	mmol/L	133 – 146
ESR	130	mm/h	0 – 15
CRP	6	mg/L	<5
Creatinine	117	µmol/L	45 – 84

**Figure 1:**
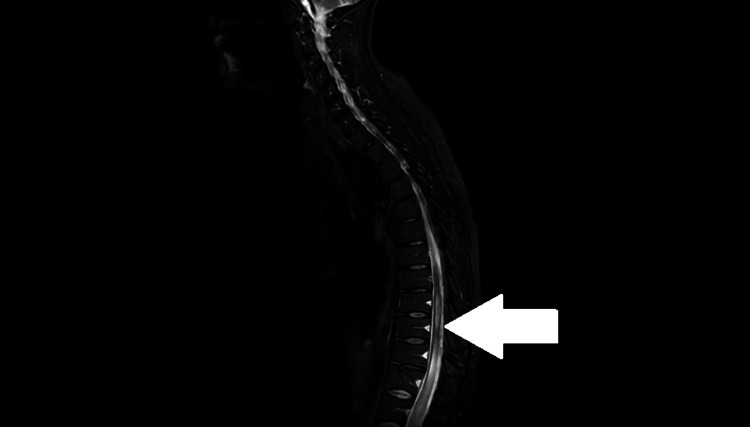
MRI spine T2-STIR sagittal sequence showing a hyperintense lesion from T9 to conus medullaris (White arrow) consistent with LETM. Sagittal T2-weighted and STIR sequences MRI of the thoracic spine demonstrates a longitudinally extensive hyperintense lesion extending from T9 to the conus medullaris (indicated by the white arrow). This imaging pattern is characteristic of longitudinally extensive transverse myelitis (LETM), a hallmark feature of NMOSD, and helps differentiate it from lupus myelitis, which typically involves shorter cord segments.

9MRI of the spine with contrast revealed a hyperintense lesion on T2/short tau inversion recovery (STIR) extending from T9 to the conus medullaris, compatible with LETM (Figure 1).

**Figure 2:**
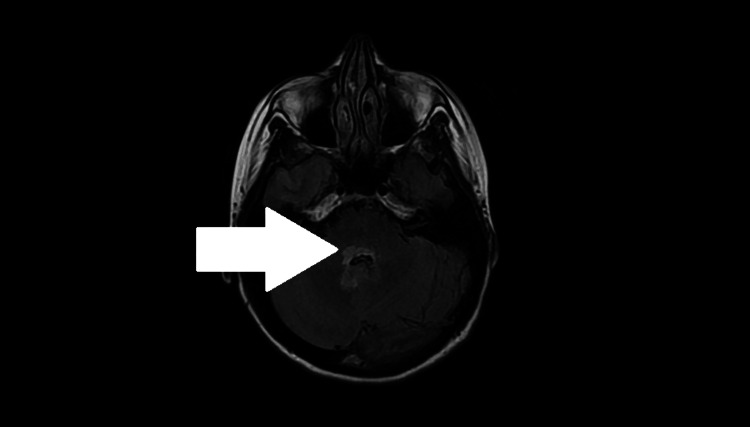
MRI brain FLAIR image showing dorsal pontine lesion with contrast enhancement (White arrow), demonstrating pontine involvement in NMOSD. Axial T2-weighted fluid-attenuated inversion recovery (FLAIR) MRI of the brain reveals a hyperintense lesion within the dorsal pons and cerebellar peduncles, with patchy contrast enhancement (white arrow). These findings correlate with the patient’s cranial nerve deficits, including diplopia and facial weakness, consistent with “eight-and-a-half syndrome.” Brainstem involvement is a recognized feature of NMOSD and helps distinguish it from isolated lupus myelitis.

**Figure 3:**
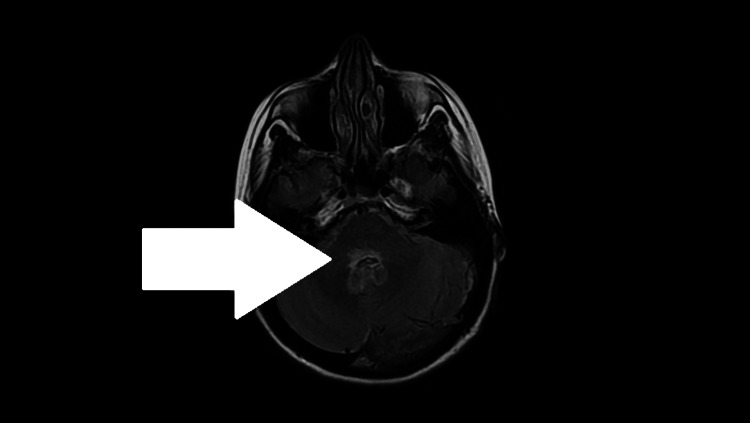
MRI brain FLAIR sequence with contrast enhancement demonstrating pontine involvement in NMOSD. Axial T2-weighted fluid-attenuated inversion recovery (FLAIR) MRI of the brain with contrast shows a hyperintense lesion within the dorsal pons and cerebellar peduncles (white arrow). That highlights active inflammation of the brainstem consistent with neuromyelitis optica spectrum disorder (NMOSD). These findings correlate with the patient’s cranial nerve deficits, including diplopia and facial weakness (8½ syndrome). Brainstem involvement is a recognized feature of NMOSD and helps distinguish it from isolated lupus myelitis.

MRI of the brain showed a hyperintense lesion within the dorsal pons and cerebellar peduncles with patchy contrast enhancement and restricted diffusion (Figures 2-3). 

Cerebrospinal fluid (CSF) analysis demonstrated clear fluid with 478 white cells/µL (80% lymphocytes), protein 0.75 g/L, glucose 2.7 mmol/L, and lactate 3.9 mmol/L (Table 2). CSF cultures and viral PCR results were negative. Serological testing for aquaporin-4 (AQP4) and myelin oligodendrocyte glycoprotein (MOG) antibodies was later performed, and both returned negative.

**Table 2:** Cerebrospinal fluid (CSF) analysis This table presents cerebrospinal fluid findings demonstrating marked lymphocytic pleocytosis and elevated protein levels, consistent with inflammatory demyelinating pathology. Normal glucose and mildly elevated lactate further support an autoimmune rather than infectious etiology, aligning with NMOSD pathology. NMOSD: neuromyelitis optica spectrum disorder

Parameter	Result	Units	Reference Range
White blood cells	478	/µL	0 – 5 /µL
Lymphocytes	80	%	< 70%
Protein	0.75	g/L	0.15 – 0.45 g/L
Glucose	2.7	mmol/L	2.2 – 4.0 mmol/L
Lactate	3.9	mmol/L	1.1 – 2.4 mmol/L

Despite seronegativity, the combination of LETM (T9 to conus) and pontine lesions, together with the clinical picture, supported a diagnosis of seronegative NMOSD overlapping with SLE. The patient was treated with intravenous methylprednisolone (1 g daily for five days) alongside acyclovir 10 mg/kg TID (renal-adjusted) as empirical antiviral coverage. Deflazacort was withheld during IV steroid therapy, and she continued hydroxychloroquine and labetalol. Supportive management included bowel care, VTE prophylaxis, and physiotherapy. After four days of treatment, her diplopia improved slightly, but lower limb paralysis persisted. She was subsequently transferred to a tertiary neurology center for plasma exchange (PLEX) and further workup.

## Discussion

This case illustrates the diagnostic complexity and clinical overlap between SLE-associated myelitis and neuromyelitis optica spectrum disorder. Both entities can present with acute spinal cord dysfunction and share radiological features, yet they differ markedly in underlying mechanisms and therapeutic response. In SLE, spinal cord injury may result from vasculitis, thrombosis, or direct antibody-mediated inflammation, whereas NMOSD is primarily an astrocytopathic process driven mainly by anti-AQP4 antibodies [7]. The identification of longitudinally extensive spinal lesions, particularly when accompanied by brainstem involvement, should raise suspicion for NMOSD even in patients with a prior lupus diagnosis or negative anti-AQP4 antibody test.

In this patient, MRI revealed an extensive T2 hyperintense lesion from T9 to the conus medullaris with concurrent pontine lesions, explaining her diplopia and facial weakness consistent with 8½ syndrome. Such combined spinal and brainstem involvement is well documented in NMOSD and rarely seen in isolated lupus myelitis [8]. The normal inflammatory markers and lymphocytic cerebrospinal fluid (CSF) findings further supported an autoimmune demyelinating process rather than an infectious etiology. Although AQP4 and MOG antibody assays were negative, the radiological pattern and clinical course fulfilled current diagnostic criteria for seronegative NMOSD [3,9]. Early initiation of high-dose intravenous methylprednisolone followed by PLEX aligns with current NMOSD management recommendations, which emphasize rapid immunosuppression to limit astrocyte damage and prevent relapse [9].

The temporal relationship between her hormonal treatment and disease onset suggests that reproductive and endocrine factors may have contributed to immune dysregulation. Estrogen and gonadotropin stimulation have been linked to heightened B-cell activity and cytokine production, potentially triggering autoimmune exacerbations in predisposed individuals [10,11]. Several reports have described similar NMOSD flares during or after pregnancy, reinforcing the link between hormonal shifts and disease activity [11].

Differentiating lupus myelitis from NMOSD has important therapeutic implications. While lupus myelitis may respond to corticosteroids and cyclophosphamide, NMOSD often requires escalation to B-cell-depleting agents such as rituximab or complement inhibition to prevent recurrence [9,12]. The decision to initiate PLEX in this patient was appropriate, given her incomplete response to corticosteroids and the suspicion of NMOSD. Continued immunosuppressive therapy and multidisciplinary follow-up involving neurology, rheumatology, and reproductive medicine teams are essential to optimize outcomes and reduce relapse risk.

Overall, this case underscores the importance of maintaining a high index of suspicion for NMOSD in patients with SLE presenting with LETM, particularly when associated with cranial nerve deficits or recent hormonal events. Early MRI evaluation, CSF analysis, and prompt immunotherapy can significantly influence neurological recovery and long-term prognosis.

## Conclusions

This case highlights a unique presentation of NMOSD in a woman with pre-existing SLE, following a recent hormonal and reproductive event. The patient initially presented with urinary retention and leg weakness, later developing diplopia and facial palsy. MRI and CSF studies confirmed the diagnosis of LETM with pontine involvement, consistent with NMOSD. Her partial response to high-dose corticosteroids and subsequent referral for PLEX underscores the importance of prompt recognition and escalation of immunotherapy. At the time of transfer, her vision had improved, and systemic symptoms stabilized, though lower limb weakness persisted. This case underscores the need for multidisciplinary management in patients with overlapping autoimmune disorders and emphasizes vigilance for neurological manifestations in SLE, especially following immunological or hormonal triggers.
